# Prediction of pancreatic neuroendocrine tumor grading using an artificial intelligence–based video analysis model (GradAINet) applied to contrast-enhanced EUS videos

**DOI:** 10.1097/eus.0000000000000190

**Published:** 2026-07-21

**Authors:** Matteo Tacelli, Adrien Meyer, Gaetano Lauri, Armine Vardazaryan, Paolo Biamonte, Bastien Andlauer, Rubino Nunziata, Gabriele Capurso, Leonardo Sosa-Valencia, Nicolas Padoy, Paolo Giorgio Arcidiacono

**Affiliations:** 1 Pancreato-Biliary Endoscopy and Endosonography Division, Pancreas Translational and Clinical Research Center, IRCCS San Raffaele Scientific Institute, Milan, Italy; 2 IHU-Strasbourg, Institute of Image-Guided Surgery, Strasbourg, France; 3 CNRS, INSERM, ICube, UMR7357, University of Strasbourg, Strasbourg, France; 4 Vita-Salute San Raffaele University, Milan, Italy.

**Keywords:** deep learning, pancreatic neuroendocrine neoplasms, predictive model, risk stratification

## Abstract

**Background and Objectives:**

Pancreatic neuroendocrine neoplasms (PNENs) are rare tumors with heterogeneous outcomes. Tumor grading (G), based on mitotic count and Ki-67 index, is the main prognostic factor guiding treatment. EUS–guided fine-needle aspiration/biopsy is the current standard but shows a misgrading rate up to 25%. We evaluated an artificial intelligence–based video analysis model to predict PNEN grading from contrast-enhanced EUS (CE-EUS) recordings.

**Methods:**

This retrospective study was conducted at Istituti di Ricovero e Cura a Carattere Scientifico San Raffaele Hospital, Milan, a European Neuroendocrine Tumor Society Center of Excellence. Patients were eligible if CE-EUS videos ≥1 minute (arterial and venous phases) and cyto-histological confirmation of PNEN were available. Exclusion criteria included mixed neuroendocrine–non-neuroendocrine neoplasms, missing Ki-67 grading, or poor video quality. CE-EUS videos were processed with a deep-learning video transformer model (GradAINet). The dataset was split into training (70%), validation (10%), and testing (20%) cohorts. Diagnostic performance was evaluated using sensitivity, specificity, positive predictive value, negative predictive value, accuracy, and F1-score.

**Results:**

Between 2022 and 2024, 115 patients were included (49 female, 42.6%): 70 had G1, and 45 had G2–G3 tumors. Overall, 253,751 video frames were analyzed. GradAINet achieved a sensitivity of 0.817 (95% confidence interval [CI]: 0.556–1.000), specificity 0.806 (95% CI: 0.588–1.000), positive predictive value 0.759 (95% CI: 0.500–1.000), negative predictive value 0.856 (95% CI: 0.667–1.000), and accuracy 0.811 (95% CI: 0.654–0.962).

**Conclusion:**

This artificial intelligence–driven CE-EUS video model shows high accuracy for PNEN grading and potentially complements EUS-guided fine-needle aspiration/biopsy. As the first video-based rather than static-image model, it represents a methodological advance. Multicenter validation on larger cohorts is needed before clinical implementation.

## INTRODUCTION

Pancreatic neuroendocrine neoplasms (PNENs) are rare and biologically distinct tumors arising from the endocrine cells of the pancreas, accounting for approximately 1%–2% of all pancreatic malignancies.^[1,2]^ Over the past few decades, their reported incidence has been steadily increasing, likely due to advancements in diagnostic modalities and heightened clinical awareness.^[3,4]^

While the diagnostic evaluation of PNENs traditionally relies on computed tomography (CT), magnetic resonance imaging (MRI), and somatostatin receptor imaging, preferably with positron emission tomography (PET)/CT, EUS has demonstrated superior sensitivity, detecting approximately 26% more PNENs than CT.^[5,6]^ Sonographically, PNENs typically appear as well-defined, round or oval, hypoechoic lesions with homogeneous echotexture and hypervascularity.^[7]^ However, detection may be challenging, as some lesions appear iso- or slightly hypoechoic, potentially mimicking other pancreatic masses such as pancreatic ductal adenocarcinoma, autoimmune pancreatitis, or cystic neoplasms.^[8,9]^

The key prognostic factor for PNENs is histological grading, based on mitotic index or Ki-67 immunostaining according to the World Health Organization (WHO) classification.^[10]^ Grade 1 asymptomatic nonfunctional PNENs <2 cm are usually managed with surveillance, whereas G2–G3 tumors are associated with poorer outcomes and require surgery or more aggressive therapies.^[11,12]^ Thus, accurate grading at diagnosis is essential for treatment planning. EUS-guided fine-needle aspiration/biopsy (EUS-FNA/B) is the current standard, providing both diagnostic and prognostic information, but distinguishing G1 from G2–G3 remains challenging.^[13,14]^ Concordance with surgical grading is variable, with a recent meta-analysis reporting 80.3% agreement and a higher risk of undergrading than overgrading (14.7% *vs.* 3.5%, *P* < 0.001).^[15,16]^ To improve diagnostic accuracy, contrast-enhanced EUS (CE-EUS) has been introduced, allowing better lesion characterization and assessment of tumor behavior.^[17]^

Well-differentiated, low-grade PNENs typically show early hyperenhancement with rapid washout, reflecting higher microvascular density (MVD), whereas high-grade tumors display reduced microvascular density and hypoenhancement, often mimicking pancreatic ductal adenocarcinoma.^[18–20]^ However, CE-EUS interpretation is subjective and operator-dependent.

Artificial intelligence (AI) may overcome this limitation and, while most studies rely on static images, EUS is a dynamic examination, performed at 24 frames per second.^[21]^

Video-based AI analysis could better exploit spatio-temporal vascular patterns. The present study evaluates, for the first time, an AI-driven CE-EUS video model for preoperative PNEN grading, potentially providing a transformative tool for tumor characterization and risk stratification.

## PATIENTS/MATERIAL AND METHODS

### Study Population

This retrospective observational study analyzed a prospectively maintained database of images/videos of patients who underwent EUS at the tertiary referral center, San Raffaele Hospital, after imaging findings on CT or MRI that were suspicious for PNENs. Patients were enrolled after approval by the institutional review board (NCT06344507).

All patients included in the study underwent EUS for the evaluation of PNENs at San Raffaele Hospital. Eligibility criteria included patients older than 18 years who had undergone CE-EUS with Sonovue (Bracco Imaging, Milan, Italy), with video recordings of at least 1 minute covering both the arterial and venous phases. Additionally, a confirmed surgical or EUS-FNA diagnosis of PNEN, with available Ki-67 immunostaining, was required. When both surgical and cytological specimens were available, tumor grading was based on surgical pathology, which was considered the diagnostic gold standard and used as ground truth for AI training. For patients who did not undergo surgery, grading was based on the Ki-67 index obtained from EUS-FNA cytological samples, which served as a surrogate reference standard.

Exclusion criteria included mixed neuroendocrine–non-neuroendocrine neoplasms, unavailable cyto-histological grading, specifically those lacking Ki-67 index assessment, poor-quality video recordings, lack of signed informed consent, and neuroendocrine tumors (NETs) of nonpancreatic origin. Patients who did not meet all inclusion criteria or failed to provide consent for data collection were excluded from the analysis.

### EUS Procedure

EUS examinations were performed by experienced endosonographers, each with a minimum annual volume of 250 pancreatic EUS procedures, using a linear echoendoscope (EG3870UTK or EG38-J10UT, Pentax Hamburg GmbH, Hamburg, Germany) connected to the Fujifilm Arietta 750 ultrasound platform. All the suspicious PNEN lesions were studied with the use of CE-EUS.

During CE-EUS, 5 mL of the microbubble contrast agent SonoVue was injected intravenously, followed by a 10 mL saline flush. This dose was selected to ensure stable and homogeneous enhancement throughout the arterial and venous phases of the CE-EUS examination.^[22]^ A continuous video recording of the contrast sequence was acquired for at least 1 minute after contrast administration, to cover both the arterial and the venous phases. All recorded videos were subsequently reviewed by 2 independent endosonographers to assess lesion enhancement characteristics and eligibility for the study.

EUS-FNA was performed using the slow-pull technique with either Franseen or Menghini needles (25 or 22 Gauges) according to the endoscopist preference. The number of passes was determined by the acquisition of a diagnostically adequate sample, as confirmed by rapid on-site evaluation performed by an expert cytotechnologist. The samples were processed using the cell-block technique for further histopathological analysis. EUS-FNA specimens were fixed in formalin, embedded in paraffin, and cut into 5-µm sections. Immunohistochemical staining for Ki-67 was performed using the MIB-1 antibody clone (1:160; Dako Corporation, Carpinteria, CA, USA). The Ki-67 proliferative index was determined by calculating the percentage of positively stained cells within 500 adjacent tumor cells in the most reactive area of the specimen. PNENs were classified according to the 2019 WHO grading system. Well-differentiated NETs were graded based on mitotic count and Ki-67 index: grade 1 (G1) for Ki-67 <3% and/or <2 mitoses per 10 high-power fields (HPF), grade 2 (G2) for Ki-67 between 3% and 20% and/or 2–20 mitoses per 10 HPF, and grade 3 (G3) for Ki-67 >20% and/or >20 mitoses per 10 HPF. Poorly differentiated neuroendocrine carcinomas were classified as high-grade tumors (G3), exhibiting aggressive behavior with a high mitotic rate and often extensive necrosis.

In patients who underwent both EUS-FNA and surgical resection, concordance analysis between cytological and surgical grading was performed. A mismatch was defined as a discordance in tumor grade between EUS-FNA cytology and final surgical histology according to the 2019 WHO classification. Cases in which the Ki-67 index could not be determined from the EUS-FNA specimen were classified as diagnostic failures and considered as false negatives.

### EUS Video Processing and Artificial Intelligence Analysis

After data anonymization, removing all sensitive patient information such as name, surname, examination date and time, and any other parameters potentially interfering with video processing, 2 expert EUS specialists manually delineated both the spatial and temporal Regions of Interest (RoI) using the MOSaiC annotation platform.^[23]^ Spatial RoIs were defined using bounding boxes encompassing the lesion and adjacent normal pancreatic parenchyma. The lesion RoI included the entire visible tumor margin while avoiding major vessels and imaging artifacts.

Temporal variables were independently annotated based on contrast enhancement dynamics to identify the arterial and venous phases of contrast enhancement. Consistent with contrast-enhanced ultrasound methodological literature, the arterial phase was defined approximately between 10 and 30 seconds after contrast injection, while the venous phase corresponded to the later enhancement period between approximately 30 and 120 seconds.^[24]^ This annotation strategy ensured that the selected RoIs captured both anatomical features and dynamic vascular patterns throughout the CE-EUS contrast sequence. Any discrepancies between the 2 annotators were resolved by consensus. The anonymized videos were securely stored in a password-encrypted database and transmitted via a dedicated online platform to AI researchers at the Institute of Image-Guided Surgery (IHU Strasbourg) in France, ensuring data integrity and compliance with confidentiality standards.

Patients were then randomly divided into training (70%), validation (10%), and test (20%) cohorts for model development and validation, ensuring label distribution was preserved. The videos exhibit varying brightness gains, RoI duration, and artifacts, reflecting the partially standardized yet practitioner-dependent settings typical of real-world procedures.

### Development of AI Systems

The final aim of developing the GradAINet AI model was to perform a binary classification task: predicting the histological grade of PNENs (G1 *vs*. G2–G3) using CE-EUS video data. The model was trained using all video frames (32 frames per second) extracted from each second of the CE-EUS recordings. The ground truth for grading was based on definitive surgical histology when available, or on EUS-FNA cytology in cases where surgical specimens were not available.

AI systems were developed and trained using the Jean Zay supercomputer (GENCI–IDRIS Grant 2023-AD011013698R1). Four spatiotemporal models designed for video recognition, namely I3D,^[25]^ MViTv2,^[26]^ C3D,^[27]^ and SlowFast,^[28]^ were benchmarked using the mmAction2^[29]^ framework. Each was fine-tuned from pretrained weights on the Kinetics-400 dataset^[30]^ to adapt to the target task.

To prevent overfitting, we applied early stopping along with data augmentation techniques, including vertical and horizontal flips and random cropping. Input images were resized so that the longest side measured 256 pixels. During training, each input clip contained 32 frames uniformly sampled from the RoI of the videos. At test time, 10 clips were extracted per RoI, and final predictions were obtained through majority voting.

The best model configuration used stochastic gradient descent with a weight decay of 0.0001 and a momentum of 0.9. The learning rate was set to 0.0032, with a batch size of 16 distributed across 2 graphics processing units. Training spanned 190 epochs, including a 10-epoch warmup period. A learning rate scheduler reduced the rate by a factor of 10 at epochs 150 and 180. Hyperparameters were optimized through grid search.

Each model was trained and evaluated both with and without RoI input (i.e., on the whole video or limited to the provided RoI). The models output a continuous predictive score between 0 (G1) and 1 (G2/G3). The final model was selected based on the lowest validation loss and evaluated on an external test set for generalization assessment.

### Statistical Analysis

A receiver operating characteristic (ROC) curve was constructed to evaluate the diagnostic performance of the test. The threshold was defined by the maximum Youden index (sensitivity + specificity − 1). At this optimal point, sensitivity, specificity, positive predictive value (PPV), negative predictive value (NPV), and overall accuracy were calculated, each with corresponding 95% confidence intervals (CIs). Univariate analysis was performed to evaluate the association between clinical variables and model predictions. All statistical analyses were 2-sided, with a significance level set at *P* < 0.05. Continuous variables were reported as mean ± standard deviation or median with range, as appropriate. Categorical variables were compared using the Fisher’s exact test, while continuous variables were analyzed using the Mann–Whitney *U* test.

## RESULTS

Between 2022 and 2024, a total of 153 patients underwent EUS for suspected PNENs at San Raffaele Hospital. After applying inclusion and exclusion criteria, 38 patients were excluded either for the absence of Ki-67 evaluation in the final histological assessment (*n* = 5, 13.1%) or because of poor quality or noncomplete sequences of the videos (*n* = 33, 86.9%). Finally, 115 patients were included in the final GradAINet analysis (Table 1).

**Table 1:** Patient baseline characteristics

Characteristics	Overall (*n* = 115)	G1 (*n* = 70)	G2-G3 (*n* = 45)
Sex (female)	49 (42.6%)	27 (38.6%)	22 (48.9%)
Solid lesion (solid)	107 (93.0%)	62 (88.6%)	45 (100.0%)
Location			
- Head	37 (32.2%)	24 (34.3%)	13 (28.9%)
- Body–tail	65 (56.5%)	40 (57.1%)	25 (55.6%)
- Neck	10 (8.7%)	5 (7.1%)	5 (11.1%)
- Multifocal	3 (2.6%)	1 (1.4%)	2 (4.4%)
Diameter of the main lesion (mm, median [IQR])	20.0 [13.0-30.5]	15.0 [11.0-22.0]	30.0 [25.0-47.0]
EUS–elastography			
- Mixed	35 (30.4%)	18 (25.7%)	17 (37.8%)
- Rigid	62 (53.9%)	37 (52.9%)	25 (55.6%)
- Soft	6 (5.2%)	5 (7.1%)	1 (2.2%)
Main pancreatic duct dilation (yes)	15 (13.0%)	5 (7.1%)	10 (22.2%)
Histologic differentiation (well-differentiated)		70 (100.0%)	45 (100.0%)
Vascular infiltration (*yes*)	29 (25.2%)	10 (14.3%)	19 (42.2%)
Presence of distant metastases (yes)	24 (20.9%)	8 (11.4%)	16 (35.6%)
Functioning (yes)	12 (10.4%)	9 (12.9%)	3 (6.7%)
KI-67 at EUS-TA (mean [SD]) *n* = 98	3.94 [8.8]	1.64 [0.8]	7.4 [13.2]
KI-67 at surgery (mean [SD]) *n* = 59	3.7 [3.9]	1.5 [0.5]	5.9 [4.5]
Ki-67 nonevaluable on FNA specimens (available only on surgical specimens)s	17/115 (14.8%)		
Grading mismatch EUS-FNA *vs*. surgery	11/59 (18.6%)		
Aggressiveness* (yes)	42 (36.5%)	13 (18.6%)	29 (64.4%)

* Presence of 1 or more of the subsequent features: dilation of main pancreatic duct, ki-67 >5%, vascular invasion, diameter>4 cm, presence of distant metastases.

† Difference in grading or EUS-TA non diagnostic on Ki-67.

EUS-TA, EUS tissue acquisition; IQR, interquartile range; SD, standard deviation.

Among these 115 patients, 49 (42.6%) were female. In 59 patients (51.3%), tumor grading was assessed on the definitive surgical histological specimen after resection, while in the remaining 56 patients (48.7%), grading was based on the EUS-FNA cytological specimen. In 11 patients (18.6%), the tumor grade determined by EUS-FNA cytology differed from that obtained on the final surgical histology: in 7 cases, EUS-FNA overestimated the grade, while in 4 cases, it underestimated it. In 17 patients (14.8%), cytology confirmed the presence of a neuroendocrine neoplasm, but Ki-67 index assessment was not possible due to insufficient material. Finally, 70 tumors were classified as G1 and 45 as G2-3 (42 G2 and 3 G3).

Overall, 253751 frames were extracted from the videos for analysis. Among the 4 benchmarked spatiotemporal architectures (I3D, MViTv2, C3D, and SlowFast), evaluated both with and without predefined RoI, I3D with RoI achieved the best performance (F1-score 80.5) and was therefore selected as the final model. The corresponding F1-scores are summarized in Table 2.

**Table 2:** Test set F1-score of the benchmarked models, using the whole video clip or limited to the preselected RoI

	I3D	MViTv2	C3D	SlowFast
Without RoI	77.0	63.3	37.1	69.8
With RoI	80.5	73.0	36.6	76.4

C3D, convolutional 3D; I3D, Inflated 3D ConvNet; MViTv2, Improved Multiscale Vision Transformers; RoI, region of interest.

**Figure 1.**
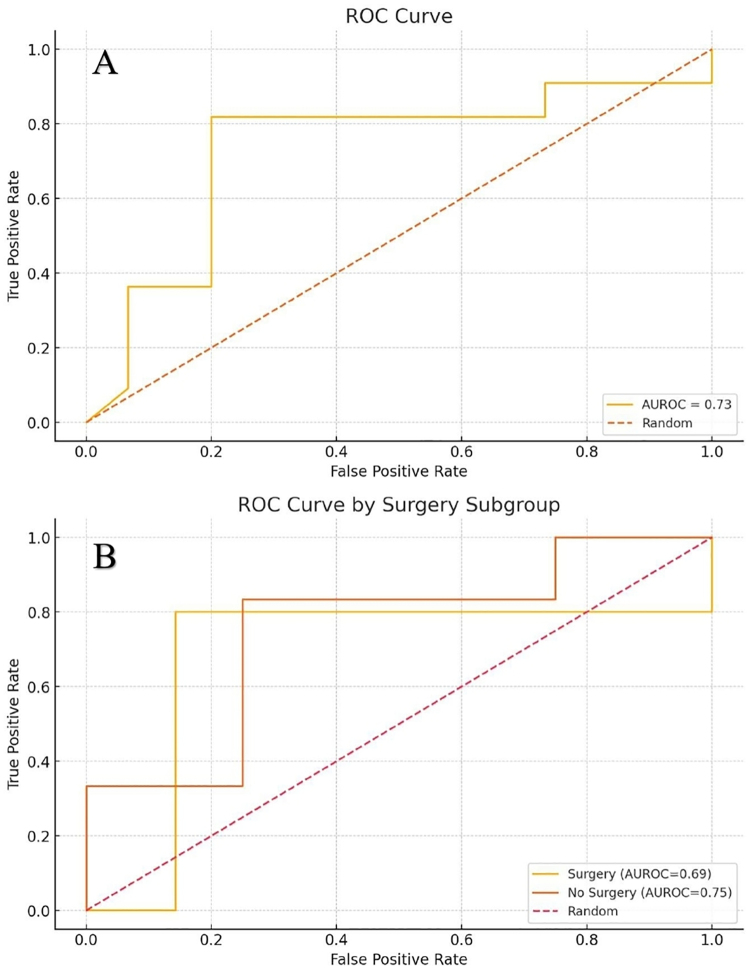
Receiver operating characteristic (ROC) curve of the GradAINet model (A) for predicting pancreatic neuroendocrine neoplasm (PNEN) grading and (B) by surgical subgroup.

The GradAINet model demonstrated robust predictive performance, with an area under the ROC curve (AUROC) of 0.73 (Figure 1A). The overall sensitivity for correct grading was 0.817 (95% CI: 0.556–1.000), specificity 0.806 (95% CI: 0.588–1.000), PPV 0.759 (95% CI: 0.500–1.000), NPV 0.856 (95% CI: 0.667–1.000), and overall accuracy 0.811 (95% CI: 0.654–0.962).

In the subgroup of patients who underwent surgical resection (*n* = 59, 51.3%), with histological grading on surgical specimens considered as gold standard, the GradAINet model achieved an AUROC of 0.69, with a sensitivity of 0.796, specificity 0.817, PPV 0.859, NPV 0.741, and accuracy 0.805. In patients without surgery, in whom grading was based on EUS-FNA, the AUROC was 0.75 (Figure 1B).

No statistically significant associations were found between the GradAINet model predictions and the clinical, pathological, or imaging variables analyzed (Table 3).

**Table 3:** Univariate analysis to predict possible factors influencing the accuracy of the GradAINet model

Variable	*P* value
Metastases site (liver *vs*. lymph nodes/ other)	0.0608
Main pancreatic duct dilation	0.1074
TNM staging	0.1188
Lesion size	0.2549
Functioning	0.3577
“Aggressiveness”	0.3988
Histologic differentiation	0.3729
Morphologic appearance (solid *vs*. solid-cystic)	0.5286
Ki-67 value	0.5668
Location in the pancreas	0.6931
Vascular infiltration	0.7096
Elastographic EUS pattern	0.8109
Presence of metastases	0.8633
Sex	0.9028

TNM, tumor node metastasis.

## DISCUSSION

This study demonstrates that the GradAINet model, trained on over 250,000 CE-EUS frames, achieved a high diagnostic performance in predicting PNEN grading, with an overall accuracy of 81.1%, sensitivity of 81.7%, and NPV of 85.6%.

Accurate preoperative grading is crucial for prognosis and treatment planning, but EUS-guided tissue acquisition often provides insufficient material, and concordance with surgical specimens is limited (80.3%), with undergrading occurring more frequently than overgrading (14.7% *vs*. 3.5%).^[15]^ In our cohort, grading was not feasible in 17 patients (14.8%), and among 59 resected cases, a mismatch was observed in 11 (18.6%). These findings highlight the limitations of EUS-based methods and the need for complementary techniques to improve grading precision.

Importantly, the availability of a surgical subgroup with definitive histological grading also allowed an indirect comparison framework between cytology-based grading and AI prediction. Within this subgroup, discrepancies between EUS-FNA cytology and surgical histology, as well as cases with insufficient material for Ki-67 assessment, further illustrate the limitations of tissue-based grading. These findings support the potential value of complementary imaging-based approaches, including AI-assisted CE-EUS video analysis, for improving preoperative tumor characterization.

CE-EUS has been proposed to refine tumor characterization and support the identification of aggressive tumors,^[31]^ but its interpretation remains subjective, a limitation that AI could potentially overcome. In fact, the rapid advancement of AI has opened new frontiers in the detection and classification of PNENs.

Previous radiomics studies using abdominal ultrasound, MRI, or CT have reported only moderate accuracy and limited generalizability.^[32–34]^ Then, a convolutional neural network–based system, called iEUS, has been developed to enhance lesion identification and differentiate PNENs from other pancreatic pathologies through EUS-based radiomics models.^[35]^ Additionally, a validated nomogram has been introduced to predict tumor grading based on EUS images, demonstrating high accuracy in the differentiation of PNEN G1 from PNEN G2/G3.^[32]^

This study represents the first attempt to integrate deep-learning algorithms with EUS video analysis containing both arterial and venous phases, rather than static images, for the prediction of PNENs grading.

Unlike conventional predictors such as tumor node metastasis stage, Ki-67, or lesion size, no clinical or pathological variables correlated with model output, suggesting that GradAINet captures complex spatiotemporal vascular features that may escape conventional human interpretation or basic statistical modeling.

Despite its strong performance, several limitations must be acknowledged. First, while our study included a large number of EUS videos, the dataset used to train the AI model was derived from a single institution, potentially limiting generalizability. Future work should focus on external validation in multicentric cohorts to confirm the reproducibility of our results across different EUS settings. Second, variability in EUS acquisition parameters, including gains and focuses of the images, might introduce bias in video interpretation. Although no formal harmonization of acquisition parameters was performed, the model was trained on real-world CE-EUS videos acquired under routine clinical conditions, which may partially enhance robustness to inter-examination variability. Nevertheless, the potential impact of acquisition heterogeneity cannot be completely excluded, and standardization of these parameters will be important for optimizing AI-based analysis in clinical practice.

Third, although the model showed encouraging performance, the external test set was relatively small (*n* = 23), resulting in wide CIs for sensitivity and specificity estimates. This limited sample size may affect the stability of performance metrics and warrants cautious interpretation of the results. Larger independent validation cohorts will be necessary to better assess the generalizability of GradAINet across different institutions and acquisition settings. In conclusion, GradAINet represents the first deep-learning approach applied to CE-EUS videos for PNEN grading, providing a novel and reliable tool for preoperative risk stratification. Future refinements may come from integrating clinical, biological, and molecular data, including genetic and transcriptomic profiles, with imaging-based AI to further enhance the assessment of tumor aggressiveness.

## Source of Funding

This research was partially supported by the ARC Foundation via the APEUS project and by French state funds managed by the ANR under grants ANR-10-IAHU-02 and ANR-21-RHUS-0001 DELIVER. The work of G.L. was supported by the European Union—Next Generation EU, Mission 4, Component 1 CUP D42B24000790004.

## Ethical Statements

Patients were enrolled after approval by the institutional review board (NCT06344507). Informed consents were obtained.

## Conflicts of Interest

Paolo Giorgio Arcidiacono is an Associate Editor of the journal. The article was subjected to the standard procedures of the journal, with a review process independent of the editor and his research group. The other authors declare no conflict of interest with regard to the content of this report.

## Author Contributions

Substantial contributions to the conception or design of the work: Matteo Tacelli, Adrien Meyer, Bastien Andlauer, Gabriele Capurso, Leonardo Sosa-Valencia, Nicolas Padoy, Paolo Giorgio Arcidiacono. Acquisition, analysis, or interpretation of data for the work: Paolo Biamonte, Armine Vardazaryan, Gaetano Lauri, Rubino Nunziata. Drafting the manuscript: Gaetano Lauri, Matteo Tacelli, Gabriele Capurso. Revising it critically for important intellectual content: All authors. Final approval of the version to be published: All authors. Accountability for all aspects of the work: All authors

## Data Availability Statement

The data supporting the findings of this study are available from the corresponding authors upon reasonable request.
